# The Strain Transfer Mechanism of Fiber Bragg Grating Sensor for Extra Large Strain Monitoring

**DOI:** 10.3390/s19081851

**Published:** 2019-04-18

**Authors:** Li Sun, Chuang Li, Chunwei Zhang, Tianqi Liang, Zihao Zhao

**Affiliations:** 1 School of Civil Engineering, Shenyang Jianzhu University, Shenyang 110168, China; leechuang1990@163.com (C.L.); 13898841197@163.com (T.L.); zhaozihao_47@163.com (Z.Z.); 2 School of Civil Engineering, Qingdao University of Technology, Qingdao 266033, China; zhangchunwei@qtech.edu.cn

**Keywords:** wide-range FBG sensor, extra-large strain monitoring, strain transfer, linearity, stability

## Abstract

This research focuses on a desensitization method to develop a wide-range FBG sensor for extra-large strain monitoring, which is an essential requirement in large scale infrastructures or for some special occasions. Under appropriate hypotheses, the strain transfer distribution of wide-range FBG sensor based on the shear-lag theory is conducted to improve the accuracy of extra-large strain measurements. It is also discussed how the elastic modulus of adhesive layer affects the strain transfer rate. Two prototypes in different monitoring ranges are designed and fabricated by two layers of steel pipe encapsulation. The presented theoretical model is verified by experimental results. Moreover, it is demonstrated that experimentation in regards to the calibration of the wide-range FBG sensor, improved the amplification coefficient up to 2.08 times and 3.88 times, respectively. The static errors are both calculated and analyzed in this experiment. The wide-range FBG strain sensor shows favourable linearity and stability, which is an excellent property of sensors for extra-large strain monitoring.

## 1. Introduction

Most structural disasters can be avoided if the structural behaviour had only been inspected, monitored, alerted, and analyzed continuously. Structural health monitoring refers to the use of non-destructive monitoring methods on-site to obtain structural response characteristics and internal mechanical information for safety assessment and damage diagnosis [1,2]. Kalkan [3] conducted the deformation and strain monitoring of the Ataturk Dam to focus on the dam’s mechanical behaviour for a period of more than 10 years. Xia [4] presented a new method for calculating structural deformation using real-time strain data, which was then used to calculate the horizontal displacement and tilt angle of a super-tall structure. Lv [5] developed a Fiber Bragg grating-based wire-pull bidirectional displacement sensor, and monitored the deformation of foundation pit of Beijing Daxing International Airport in real time. Alexander [6] embedded electrical strain gages to monitor the strain and deflection state of glass fiber-reinforced polymer. In this regard, strain and deformation monitoring has played an important role in health monitoring of civil and industrial structures, especially for strain monitoring of special structures where it is supremely important that early warning signs are established. For instance, it can be used to determine pre-stress, propagation of cracks, leakage in the tunnel, gross distortion of frames, and stress concentration in the steel. It is therefore necessary to develop a strain and deformation monitoring sensor of wide-sensing range to satisfy the need of special industrial projects to replace traditional sensors which are made of potentiometer and capacitance, etc. For example, a conventional extensometer is used for extra-large strain measurement, which is converted from the macroscopic displacement between two points of a component so the accuracy cannot be ignored. Narendiran [7] developed a wide-range of capacitive sensors that were suitable for measuring both linear and angular displacements, which consisted of a cylindrical shaft with a semi-hollow cylinder attached in the center. The prototype sensor had a linear displacement range of (−40 to 40 mm) and an angular displacement range of (0–360°). For the large crack monitoring application, Duan [8] presented the fabrication of an MEMS-based capacitive wide-scale strain sensor. The change in strain caused a measurable change in voltage for the signal by the capacitance sensor. The measured large-scale strain range in his work was between 0 and 20,000με. Johnson [9] presented a comprehensive analysis of a polystyrene foam subjected to large strains and high pressures based on the experimental data, which was obtained from uniaxial stress tests in compression with compressive strains of almost 50% by embedding or penetrating CNTs onto the surface layer of TPU multifilaments. Qin [10] obtained high conductivity of CNTs–TPU fibers and presented that the changes in resistance were highly reversible under cyclic stretching up to a strain deformation of 400%. As of now, the conventional sensors of a wide-range suffer from a limited sensitivity, low stabilization, and a complex system.

On the other hand, Fiber Bragg grating (FBG) sensors have attracted wide attentions of the mechanical research community because of their many specific characteristics such as small mass, high sensitivity, and immunity to electromagnetic interference [11,12], which are suitable for displacement, acceleration, and pressure monitoring of large-scale structural components such as dams, bridges, and the aerospace and nuclear engineering fields. Sun [13] investigated a super wide-range FBG displacement sensor based on an eccentric gear, which was applied in monitoring of the crack in a self-repairing performance test of shape memory alloy (SMA). Zou [14] presented a wide-range displacement sensor system using the FBG sensor, spring, and a twin-core fiber. The research presented quantitative analysis of the theory of the FBG–spring system, which can be used to monitor the cracks in buildings. Jiang [15] developed an FBG tilt sensor of distinguishable circumferential inclined direction with wide measuring range. To obtain the measuring characteristics, calibration experiment on one prototype of the proposed FBG tilt sensor was carried out. The sensitivities of these two-strain sensitivity FBGs were 140.85°/nm and 101.01°/nm over a wide range of 60°. Kuang [16] proposed the measurement method of large strains in geotextile sheets using plastic optical fibre (POF) sensors. Similarly, Shen [17] studied a new kind of large strain sensor based on the FBG sensor, the sensing element of which consisted of a metal trapezoidal frame and remains capable of sensing large strain of the body. Joakim [18] presented a non-contact optical method for determining the large-strain tensile behaviour of polymers using a purpose-built temperature chamber made of polycarbonate. The above research [13,14,15,16,17,18] scarcely made the comprehensive theoretical analysis so that inexplicable error appears. In order to obtain accurate monitoring results, previously presented strain transfer analysis of FBG sensor was studied by Sun [19,20]. In this paper, a wide-range FBG sensor is developed, the mechanism of which is based on how to transfer extra-large strain of matrix to a small strain of FBG sensor. The theoretical results are verified by experimental tensile tests.

## 2. Development of Wide-Range FBG Strain Sensor

**Figure 1:**
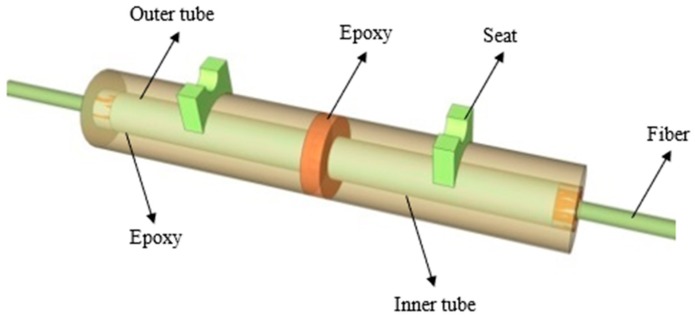
The schematic diagram of wide-range FBG strain sensor.

In this research, a wide-range FBG strain sensor is encapsulated by two layers of steel pipes, U-type bearings, bare FBG sensor, and adhesive of 353ND, etc., as shown in Figure 1. The inner steel pipe has a diameter of 1.5 mm with a length of 40 mm, whereas the outer steel pipe has a diameter of 2 mm with a length of 20 mm. The inner steel pipe works to provide fixing to the fibre, and the outer steel pipe helps in strain transfer. By adjusting the clamping length of the support and gauge length of the encapsulated FBG sensor, the mechanism of strain transfer from the extra-large strain of the matrix to small strain of FBG sensor is established. The wide-range FBG sensor breaks through the inherent range limitation and improves the measurable range. In this paper, 2 times and 4 times range of the FBG strain sensors are designed for calibration testing when the clamping length of the support (La) is 25 mm and 45 mm, respectively.

## 3. Strain Transfer Analysis

Strain transfer rate of an FBG sensor is the strain of fiber divided by the strain of matrix, which reveals the mechanism of strain transfer from extra-large strain of matrix to small strain of FBG sensor [21,22,23]. For this mechanism to work effectively, three appropriate assumptions were applied in this study to simplify the model:

(1) The material of optical fiber is linear elastic.
(2) Axial stress of the optical fiber is caused by the shear stress distributed on its surface, which results in shear deformation of the adhesive layer.
(3) There is no relative slip between the bare FBG and the adhesive layer.

**Figure 2:**
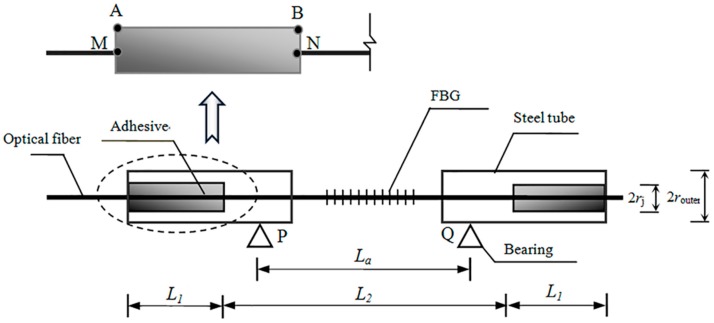
Schematic diagram of wide-range FBG sensor.

As shown in Figure 2, the computational model of a wide-range FBG sensor model is established, among which point A and B are the boundary points of the adhesive layer, whereas point M and point N are the boundary points of the optical fiber. The bare FBG sensor is bonded into two pieces of steel tubes for strain transfer, whose gap is reserved with a width of 1 mm. Where L_1_, L_2_, and L_a_ are defined as the length of the adhesive layer, gauge, and clamping bearings respectively.

**Figure 3:**
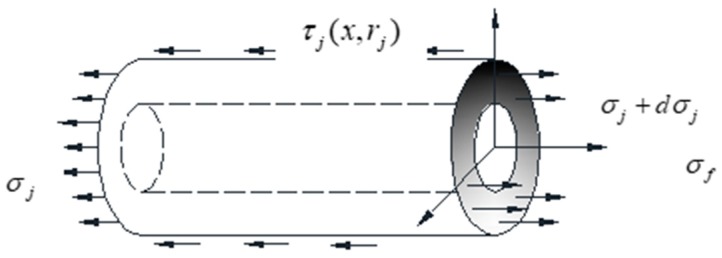
Mechanical mechanism diagram of adhesive layer micro unit.

**Figure 4:**
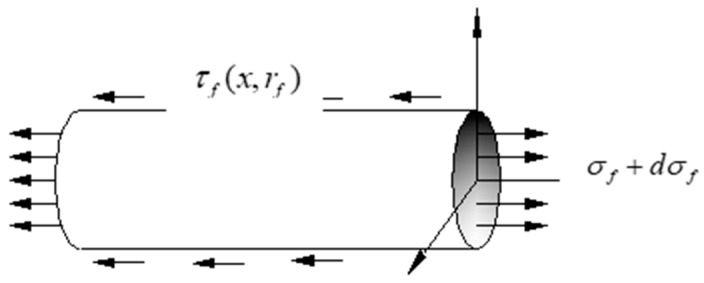
Mechanical mechanism diagram of optical fiber micro unit.

Figure 3 illustrates the mechanism of adhesive layer micro unit. Similarly, Figure 4 demonstrates the mechanism of the optical fiber micro unit, and its analysis is as follows,

(1) $${2\pi r}_{f}\times \tau_{f}(x,r_{f})dx+{\pi \times r}_{f}^{2}d\sigma_{f}=0$$

(2) $$\frac{d\sigma_{f}}{dx}=-\frac{2\tau_{f}(x,r_{f})}{r_{f}}$$

where $\sigma_{f}$, $\tau_{f}(x,r_{f})$, $r_{f}$ are the normal stress, the surface shear stress and the radius of the optical fiber respectively. From Figure 2, the following equation can be obtained:

(3) $$2\pi r_{j}\cdot \tau_{j}(x,r_{j})\cdot dx-2\pi r_{f}\cdot \tau_{f}(x,r_{f})\cdot dx+\pi (r_{j}{}^{2}-r_{f}{}^{2})\cdot d\sigma_{j}=0$$

(4) $$\tau_{j}(x,r_{j})=\frac{r_{f}}{r_{j}}\cdot \tau_{f}(x,r_{f})-\frac{r_{j}{}^{2}-r_{f}{}^{2}}{2r_{j}}\cdot \frac{d\sigma_{j}}{dx}$$

where $\tau_{j}(x,r_{j})$, $r_{j}$, $\sigma_{j}$ are the shear stress of the external surface, the radius and the normal stress of the adhesive layer, respectively. By substituting Equation (2) into Equation (4), the modified equation can be expressed as

(5) $$\tau_{j}(x,r_{j})=-\frac{r_{f}{}^{2}}{2r_{j}}\cdot \frac{d\sigma_{f}}{dx}-\frac{r_{j}{}^{2}-r_{f}{}^{2}}{2r_{j}}\cdot \frac{d\sigma_{j}}{dx}$$

(6) $$\tau_{j}(x,r_{j})=-\frac{E_{f}r_{f}{}^{2}}{2r_{j}}\cdot (\frac{d\varepsilon_{f}}{dx}+\frac{r_{j}{}^{2}-r_{f}{}^{2}}{2r_{j}}\cdot \frac{E_{j}}{E_{f}}\cdot \frac{d\varepsilon_{j}}{dx})$$

where E*_f_* and E*_j_* are the elastic modulus of optical fiber and adhesive that have the same strain rate. This leads to the following expression:

(7) $$\frac{d\varepsilon_{f}}{dx}≅\frac{d\varepsilon_{j}}{dx}$$

where *ε_f_* and *ε_j_* are the strain of the optical fiber and adhesive, respectively. Since the elastic modulus of FBG is much larger than the adhesive layer, it can be simplified as the following:

(8) $$\frac{r_{j}{}^{2}-r_{f}{}^{2}}{2r_{j}}\cdot \frac{E_{j}}{E_{f}}\cdot \frac{d\varepsilon_{j}}{dx}≅ο(\frac{d\varepsilon_{f}}{dx})$$

Substituting Equations (7) and (8) into Equation (6), the resulting equation can be written as:

(9) $$\tau_{j}(x,r_{j})=-\frac{r_{f}^{2}}{2r_{j}}\cdot \frac{d\sigma_{f}}{dx}=-\frac{r_{f}^{2}}{2r_{j}}\cdot E_{f}\cdot \frac{d\varepsilon_{f}}{dx}$$

(10) $$\tau_{j}(x,r_{j})=G_{j}\cdot \gamma (x,r_{j})≅G_{j}\cdot \frac{du}{dr_{j}}$$

where *u*, $G_{j}$, γ are the axial displacement, shear modulus, and shear strain of adhesive layer, respectively. The integral equation can be described as

(11) $$\int_{r_{f}}^{r_{i}}\tau_{j}(x,r_{j})\cdot dr_{j}=\int_{u_{M}}^{u_{A}}G_{j}\cdot du$$

(12) $$\int_{r_{f}}^{r_{i}}\tau_{j}(x,r_{j})\cdot dr_{j}=\int_{r_{f}}^{r_{i}}\left(-\frac{r_{f}^{2}}{2r_{j}}\cdot E_{f}\cdot \frac{d\varepsilon_{f}}{dx}\right)\cdot dr_{j}$$

where $u_{A}$, $u_{M}$, *r_i_* are A-point displacement of adhesive layer, M-point displacement of optical fiber and the radius of steel tube, respectively. The displacement compatibility equation can be obtained as follows:

(13) $$\begin{array}{l} u_{A}-u_{M}=-\frac{E_{f}}{2G_{j}}\cdot r_{f}^{2}\cdot \ln(\frac{r_{i}}{r_{f}})\cdot \frac{d\varepsilon_{f}}{dx}=-\frac{1}{k^{2}}\cdot \frac{d\varepsilon_{f}}{dx} \end{array}$$

(14) $$u_{A}=u_{i}\cdot \frac{L_{a}}{L_{2}{+2L}_{1}}$$

(15) $$u_{M}=u_{f}+\frac{u_{f}}{L_{1}}\cdot \frac{L_{2}}{2}$$

where $u_{i}$ and $u_{f}$ are the matrix displacement and optical fiber deformation, respectively. Among which

(16) $$G_{j}=\frac{E_{j}}{2(1+\mu )}$$

(17) $$k^{2}=\frac{1}{\left(1+\mu )\cdot \frac{E_{f}}{E_{j}}\cdot r_{f}^{2}\cdot \ln(\frac{r_{i}}{r_{f}}\right)}$$

(18) $$\frac{L_{a}}{L_{2}+2L_{1}}\cdot u_{i}-\frac{L_{2}{+2L}_{1}}{2L_{1}}\cdot u_{f}=-\frac{1}{k^{2}}\cdot \frac{d\varepsilon_{f}}{dx}$$

where, μ is the poisson ratio. By differentiating Equation (18) with respect to x, the differential equation of optical fiber strain and matrix strain can be obtained as follows:

(19) $$\frac{d\varepsilon_{f}^{2}}{dx^{2}}-\frac{(L_{2}{+2L}_{1})\cdot k^{2}}{2L_{1}}\cdot \varepsilon_{f}=-\frac{L_{a}\cdot k^{2}}{L_{2}{+2L}_{1}}\cdot \varepsilon_{i}$$

Hence, the general solution of the differential equation can be obtained through the following equation:

(20) $$\varepsilon_{f}(x)=C_{1}\cdot e^{\sqrt{\frac{L_{2}{+2L}_{1}}{2L_{1}}}\cdot kx}{+C}_{2}\cdot e^{-\sqrt{\frac{L_{2}{+2L}_{1}}{2L_{1}}}\cdot kx}+\frac{2L_{1}\cdot L_{a}}{(L_{2}{+2L}_{1}{)}^{2}}\cdot \varepsilon_{i}$$

where, $\varepsilon_{f}(x)$ and *ε_i_* are the optical fiber strain and matrix strain whereas $C_{1}$ and $C_{2}$ are the integral constants. As a result of the freedom of boundary points of the optical fiber, the following condition can be satisfied:

(21) $$\varepsilon_{f}(L_{1})=\varepsilon_{f}(-L_{1})=0$$

(22) $$C_{1}{=C}_{2}=-\frac{L_{1}\cdot L_{a}\cdot \varepsilon_{i}}{(L_{2}{+2L}_{1}{)}^{2}\cdot \cosh\left[\sqrt{\frac{L_{2}{+2L}_{1}}{2L_{1}}}\cdot k\cdot L_{1}\right]}$$

From Equations (20) and (22), the distribution rate of strain transfer can be acquired as follows:

(23) $$\alpha (x)=\frac{\varepsilon_{f}(x)}{\varepsilon_{i}}=\frac{2L_{1}\cdot L_{a}}{(L_{2}{+2L}_{1}{)}^{2}}\cdot \left\{1-\frac{\cosh(\sqrt{\frac{L_{2}{+2L}_{1}}{2L_{1}}}\cdot k\cdot x)}{\cosh(\sqrt{\frac{L_{2}{+2L}_{1}}{2L_{1}}}\cdot k\cdot L_{1})}\right\}$$

where α(x) is the strain transfer rate. The monitoring mechanism on how to transfer from extra-large strain of matrix to small strain is clarified in Equation (23). Therefore, the amplification coefficient in terms of reciprocal of strain transfer rate can be written as follows:

(24) $$\eta =\frac{1}{\alpha }=\frac{(L_{2}{+2L}_{1}{)}^{2}}{2L_{1}\cdot L_{a}\cdot \left\{1-\frac{1}{\cosh(\sqrt{\frac{L_{2}{+2L}_{1}}{2L_{1}}}\cdot k\cdot L_{1})}\right\}}$$

where *η* is the amplification coefficient of wide-range FBG sensor. Combining the above analysis, the monitoring process of extra-large strain is proposed as follows: Firstly, the strain monitoring data of bare FBG are demodulated into the wavelength data of Δλ_B_, which are acquired by the demodulator of SM-130 equipment from Micron Optics Int company in real time. Secondly, considering the influence of elastic modulus and thickness of adhesive layer, the strain transfer analysis of matrix and bare FBG is proposed using Equation (23), which is used for revising the extra-large strain of the special structure. The mechanical performance parameters of the special structural component could therefore be accurately perceived from the self-developed wide-range FBG sensor.

## 4. Theoretical Analysis Results

### 4.1. Strain Transfer Rate Distribution of Wide-Range FBG Sensor

**Figure 5:**
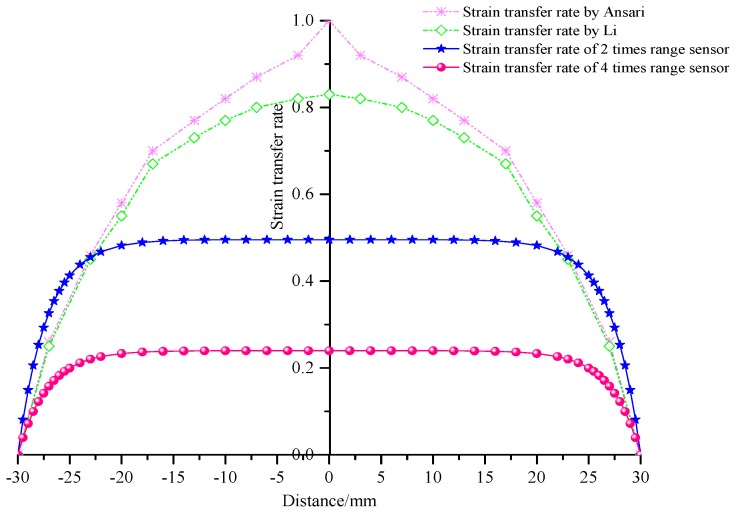
The strain distribution of wide-range FBG sensor.

Physical and mechanical parameters of fiber and adhesive are reported in Table 1:

Figure 5 provides the longitudinal strain distribution principle of wide-range FBG sensor and its strain transfer mechanism. Using Equation (23), the strain transfer rate distribution curves of 2 times and 4 times range of FBG sensors are obtained, with an interlayer length of 20 mm and a gauge length of 20 mm. In the adhesive layer range, the strain transfer rate of wide-range FBG sensor transformed from 0 to maximum. Since the sensing part of the bare FBG sensor is located in the −10 mm~10 mm interval of the coordinate system, the maximum strain transfer rate reaches 0.49 and 0.27, with a contrasting strain transfer rate of 1.00 by Ansari [24] and 0.86 by Li [25]. By reducing the sensitivity of the sensor, wide-range FBG sensors are developed to perceive extra-large strain, which, theoretically, improved the monitoring range by 2.04 times and 3.70 times, respectively.

### 4.2. Influence of Elastic Modulus on Strain Transfer Rate

**Figure 6:**
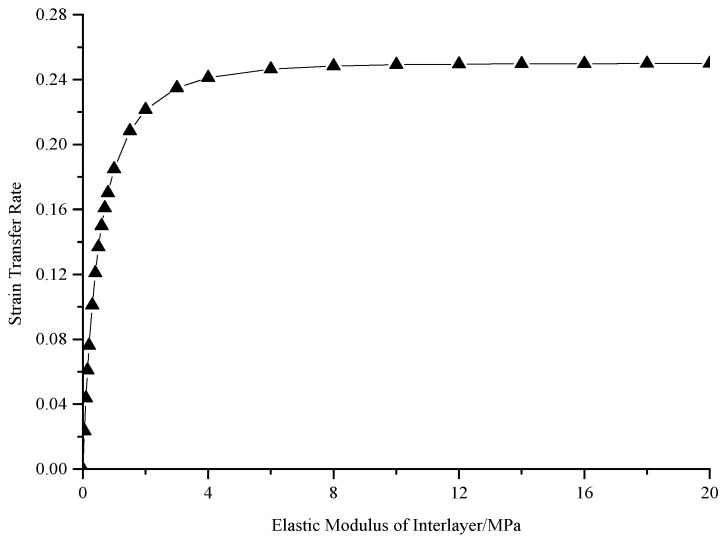
Influence of elastic modulus on strain transfer rate of wide-range FBG sensor.

The influence of elastic modulus on strain transfer rate of the FBG sensor could not be neglected, as shown in Figure 6. In the light of 4 times range of the FBG sensor, with the increase of elastic modulus of adhesive layer, strain transfer rate increases gradually, but the slope decreases accordingly. The strain transfer rate increases sharply with the increase of elastic modulus in the early stage. Until working elastic modulus is 4 MPa or greater, the strain transfer rate is stable. This analysis contributes to offer guidance on how to choose economical and reasonable elastic modulus of adhesive layer for an encapsulating wide-range FBG sensor.

## 5. Calibration Test of a Wide-Range FBG Strain Sensor

### 5.1. The Test Process

**Figure 7:**
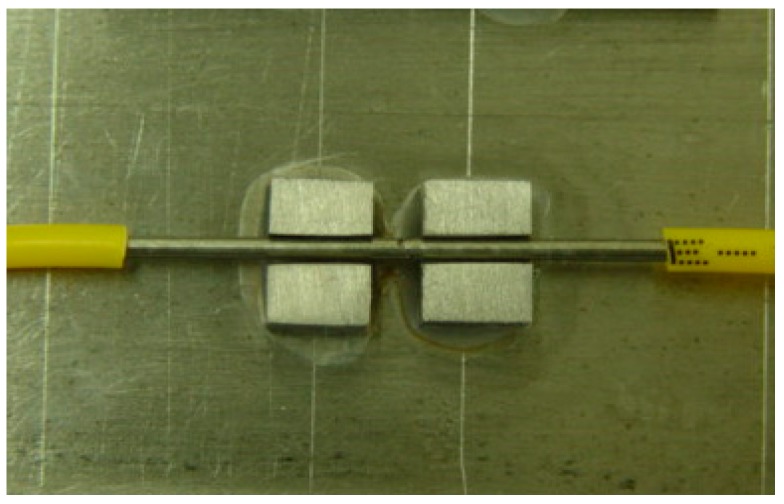
The prototype of wide-range FBG sensor.

**Figure 8:**
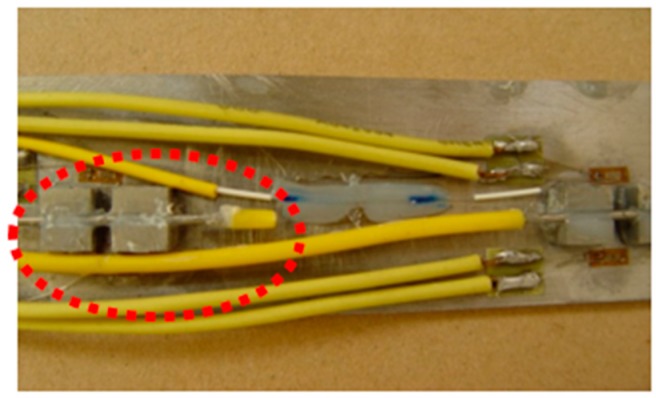
The specimen of hard aluminum.

**Figure 9:**
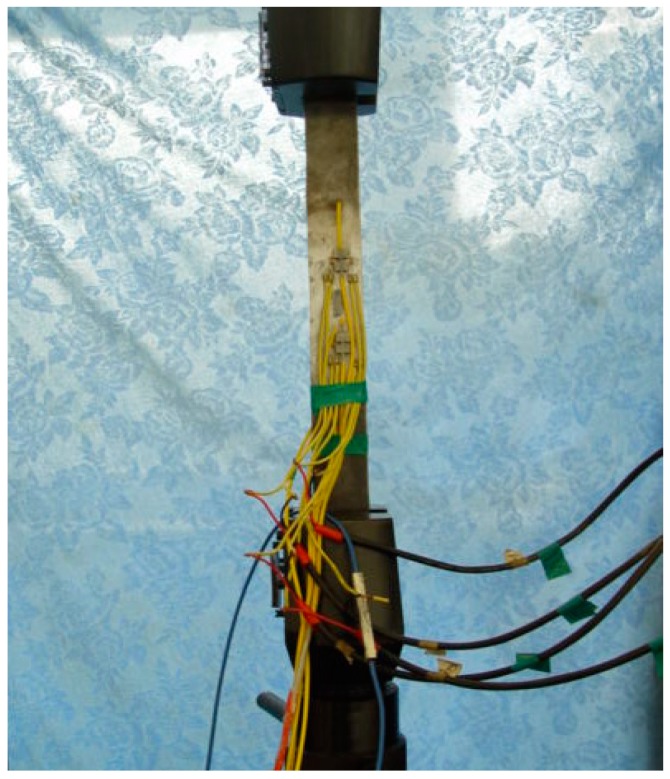
The calibration test.

As described earlier, for the calibration test, 2 times and 4 times range of FBG strain sensors are developed, which are shown from Figure 7, Figure 8 and Figure 9. In the test, wide-range FBG strain sensors are bonded on the tensile hard aluminum plate in need of sanding and alcohol wiping ahead of time. The size of the hard aluminum plate is 400 mm × 50 mm × 3 mm. The experimental equipment consists of a FBG demodulator of SM-130 used for wavelength data acquisition, resistance strain gauge of XL2101C used for strain data acquisition, and a universal testing machine of Xin-Sansi. The adopted loading process using a universal testing machine is as follows:

(1) Bare FBG strain sensor, 2 times and 4 times range of FBG strain sensors are bonded on the tensile specimen for three cyclic tests.
(2) In every cyclic test, the pre-loading applied to the steel plate is 1 kN, the step loading is 1 kN with pausing for ten seconds until the total loading reaches 51 kN.
(3) In ten seconds pause time, the strain gauge and FBG demodulator acquire the immediate data, respectively, which are recorded and analyzed as follows:

### 5.2. Analysis of the Test

**Figure 10:**
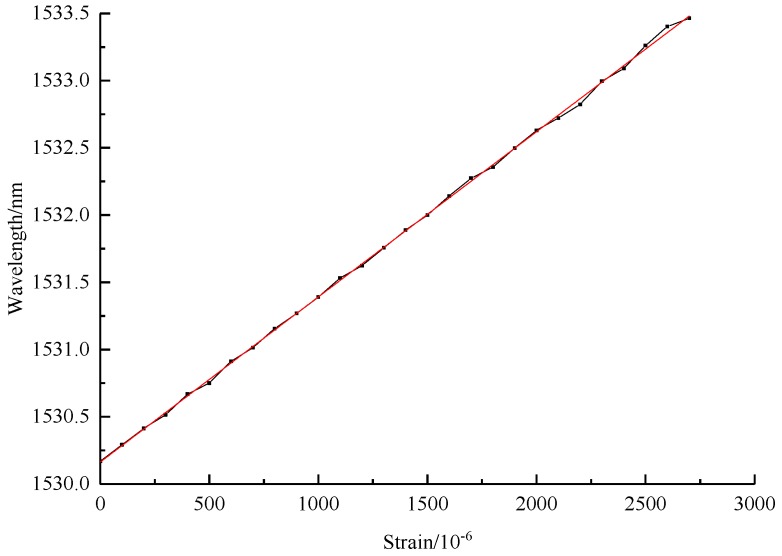
Calibration of bare FBG sensor.

**Figure 11:**
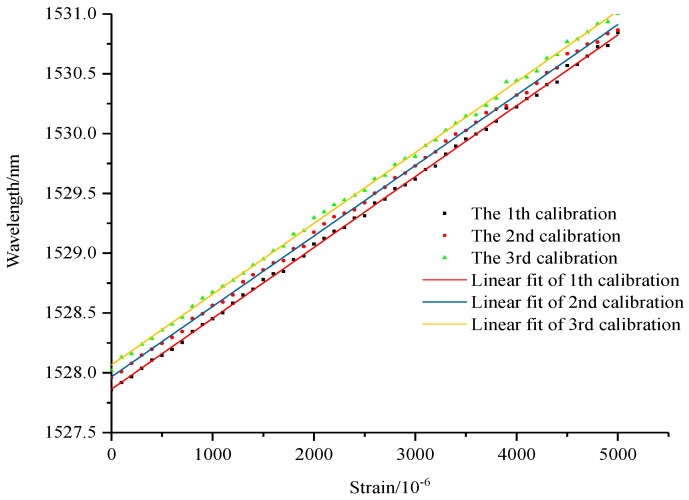
Calibration of 2 times range sensor.

**Figure 12:**
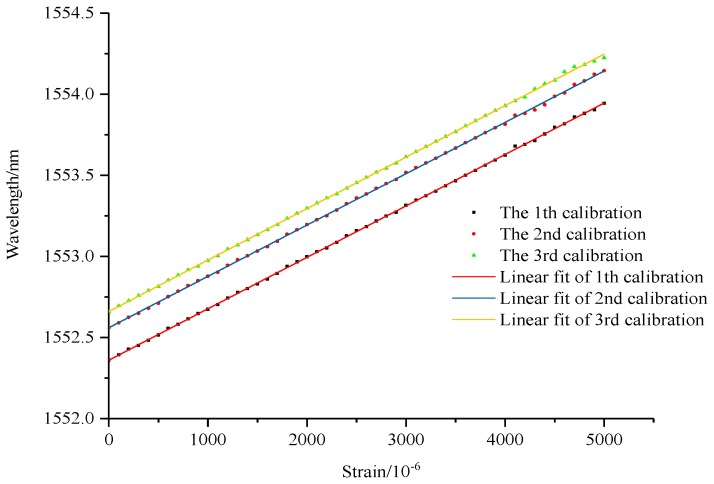
Calibration of 4 times range sensor.

The specimen of hard aluminium plate is stretched by test machine for three cycles, among which 2 times and 4 times range of FBG strain sensors could acquire data for three cycles, and the calibration results are shown in Figure 10, Figure 11 and Figure 12. However, the bare FBG sensor is broken in the first cycle of 2700μƐ, and so therefore the monitoring range is limited. As the results show, the calibration equation of bare FBG sensor is y = 1.23x/1000 + 1530.17, the sensitivity coefficient of which is 1.23 pm/μƐ. The calibration curve equation of 2 times range of FBG strain sensors are y = 0.592x/1000 + 1527.86, y = 0.589x/1000 + 1527.96 and y = 0.592x/1000 + 1528.02. Their sensitivity coefficients are 0.592 pm/μƐ, 0.589 pm/μƐ and 0.592 pm/μƐ, respectively, an average of 0.591 pm/μƐ. The calibration curve equation of 4 times range of FBG strain sensors are y = 0.317x + 1552.36, y = 0.316x + 1552.56 and y = 0.317x + 1552.66. Their sensitivity coefficients are 0.317 pm/μƐ, 0.316 pm/μƐ and 0.317 pm/μƐ, respectively, an average of 0.317 pm/μƐ. Based on the computing method of $\eta =\frac{K_{\mathrm{bare}}}{K_{1}}$, amplification coefficients of wide-range FBG strain sensors improve 2.08 times and 3.88 times experimentally, compared to the theoretical findings of 2.04 times and 3.70 times. This demonstrates errors of 2% and 4.6%, respectively.

### 5.3. Static Errors of the Wide-Range FBG Sensor

The static characteristic of the sensor refers to the relationship between the output and the input for the static input signal, including: linearity and repeatability, which are used for evaluating the accuracy of the wide-range FBG sensor monitoring.

(1) Linearity performance (*e*_L_)

Linearity is defined as a maximum difference of test results and fitting results divided by the whole output range of the test results, which is also called the nonlinear error, as shown in Equation (25).

(25) $$e_{L}=\frac{\Delta \lambda_{max}}{y_{FS}}\times 100\%=\left\{ \begin{array}{c} 2.68\%(\in 2\;times\;range\;sensor)\\ 1.3\%(\in 4\;times\;range\;sensor) \end{array}\right.$$

(2) Repeatability performance (*e*_H_)

Repeatability performance is the algebraic difference of the data obtained from several repeating measurements, which is acquired by standard deviation, as shown in Equation (26).

(26) $$e_{H}=\sigma =\sqrt{\frac{\sum_{i=1}^{n}(y_{i}-\bar{y}{)}^{2}}{n-1}}=\left\{ \begin{array}{c} 8.6\%(\in 2\;times\;range\;sensor)\\ 7.5\%(\in 4\;times\;range\;sensor) \end{array}\right.$$

(3) Static errors (*e*_s_)

By substituting *e*_L_, *e*_H_, the calculation formula of static error is as follows:

(27) $$e_{s}=\sqrt{e_{L}^{2}+e_{H}^{2}}=\left\{ \begin{array}{c} 9\%(\in 2\;times\;range\;sensor)\\ 7.6\%(\in 4\;times\;range\;sensor) \end{array}\right.$$

By calculation, the static error of 2 times range of FBG strain sensor is 9%, and that of 4 times range of FBG strain sensor is 7.6%. Through errors analysis, temperature variation, adhesive effects, and structural damage could cause some drift of sensor wavelengths in the test over time. According to the experimental results, a wide-range FBG strain sensor shows favourable linearity and stability, which makes it feasible for huge-strain monitoring.

## 6. Conclusions

This paper proposes a design method for a wide-range FBG strain sensor that can be used for extra-large strain monitoring in special structures. Considering the thickness and elastic modulus of the adhesive layer, strain transfer rate distribution of wide-range FBG strain sensor is analyzed for its effectiveness in accurate measurement. For this purpose, the 2-times and 4-times ranges of FBG sensors are developed in the calibration test. The resulting amplification coefficients are 2.08 times and 3.88 times, respectively, having errors of 2% and 4.6%, respectively, when compared with theoretical results. The static error of 2 times range of FBG strain sensor is 9%, and that of 4 times range of FBG strain sensor is 7.6%. This research proves that the proposed wide-range FBG sensor not only perceives the extra-large strain of the special structure, but also provides accurate measurement, which has promising applications in structural health monitoring of special structures.

## Figures and Tables

**Table 1:** Physical and mechanical parameters.

Parameter/Unit	Symbol	Value
Elastic modulus of optical fiber/MPa	*E_g_*	72,000
Elastic modulus of interlayer/MPa	*E_j_*	30
Poisson ratio	*µ*	0.48
Radius of optical fiber/um	*r_f_*	62.5
Radius of steel tube/mm	*r_i_*	1
Gauge ratio parameter	A	1.25
Interlayer length/mm	L_1_	20
Gauge length/mm	L_2_	20
